# Virological and immunological correlates of HIV posttreatment control after temporal antiretroviral therapy during acute HIV infection

**DOI:** 10.1097/QAD.0000000000003722

**Published:** 2023-09-11

**Authors:** Pien M. van Paassen, Lisa van Pul, Karlijn van der Straten, Ninée V.J.E. Buchholtz, Marloes Grobben, Ad C. van Nuenen, Karel A. van Dort, Brigitte D. Boeser-Nunnink, Mo D. van den Essenburg, Judith A. Burger, Matthijs van Luin, Suzanne Jurriaans, Rogier W. Sanders, Wendy T. Swelsen, Jori Symons, Michelle J. Klouwens, Monique Nijhuis, Marit J. van Gils, Jan M. Prins, Godelieve J. de Bree, Neeltje A. Kootstra

**Affiliations:** a Amsterdam UMC location University of Amsterdam, Experimental Immunology, Meibergdreef 9; b Amsterdam Institute for Infection and Immunity, Infectious Diseases; c Department of Medical Microbiology and Infection Prevention, Laboratory of Experimental Virology, Amsterdam UMC, Amsterdam; d Department of Medical Microbiology, Translational Virology, University Medical Center Utrecht; e Department of Clinical Pharmacy, Division Laboratories, Pharmacy and Biomedical Genetics, University Medical Center Utrecht, Utrecht University, Utrecht; f Department of Immunogenetics, Sanquin Diagnostic Services, Amsterdam, the Netherlands; g Department of Internal Medicine, Division of Infectious Diseases, Amsterdam UMC, Amsterdam, The Netherlands.

**Keywords:** acute HIV infection, antiretroviral therapy, CD8^+^ T cells, HIV remission, posttreatment control

## Abstract

**Objective:**

People with HIV rarely control viral replication after cessation of antiretroviral therapy (ART). We present a person with HIV with extraordinary posttreatment control (PTC) for over 23 years after temporary ART during acute HIV infection (AHI) leading to a new insight in factors contributing to PTC.

**Design/methods:**

Viral reservoir was determined by HIV qPCR, Intact Proviral DNA Assay, and quantitative viral outgrowth assay. Viral replication kinetics were determined in autologous and donor PBMC. IgG levels directed against HIV envelope and neutralizing antibodies were measured. Immune phenotyping of T cells and HIV-specific T-cell responses were analyzed by flow cytometry.

**Results:**

The case presented with AHI and a plasma viral load of 2.7 million copies/ml. ART was initiated 2 weeks after diagnosis and interrupted after 26 months. Replicating virus was isolated shortly after start ART. At 18 years after treatment interruption, HIV-DNA in CD4^+^ T cells and low levels of HIV-RNA in plasma (<5 copies/ml) were detectable. Stable HIV envelope glycoprotein-directed IgG was present during follow-up, but lacked neutralizing activity. Strong antiviral CD8^+^ T-cell responses, in particular targeting HIV-gag, were detected during 25 years follow-up. Moreover, we found a P255A mutation in an HLA-B∗44 : 02 restricted gag-epitope, which was associated with decreased replication.

**Conclusion:**

We describe an exceptional case of PTC, which is likely associated with sustained potent gag-specific CD8^+^ T-cell responses in combination with a replication attenuating escape mutation in gag. Understanding the initiation and preservation of the HIV-specific T-cell responses could guide the development of strategies to induce HIV control.

## Introduction

Despite the success of current antiretroviral therapy (ART) in suppressing viral replication, HIV is not eradicated and persists in viral reservoirs. When ART is interrupted, in the majority of individuals viral rebound will occur within several months, leading to disease progression [1]. Attempts have been undertaken to find therapeutic agents able to eradicate the rebound-competent viral reservoir (so-called ‘sterilizing cure’). A sterilizing cure has been proven possible for a few patients that underwent stem cell transplantation because of a hematological malignancy, using a donor homozygous for the CCR5-delta 32 genotype [2–5]. However, on a broad scale, a ‘functional’ cure appears more feasible. A functional cure, or ‘remission,’ is defined as durable control of virus replication in the absence of any ART. This could be either spontaneous control of HIV with a plasma viral load below 50 copies/ml in the absence of therapy (‘elite’ control), or when viral control is reached after ART interruption, that is, posttreatment control (PTC). Elite control is thought to be mainly mediated by virus-specific CD8^+^ T cells that target conserved epitopes located in vulnerable sites of viral proteins, and is associated with specific, protective HLA haplotypes, which are not found in PTC [6].

Early treatment initiation during the acute phase of the infection (AHI), resulting in a smaller viral reservoir, seems to be an important factor in achieving PTC [6–8]. This was first shown in the VISCONTI study [6]. The CHAMP study showed that PTC was more often found in early treated individuals compared with people who initiated ART in the chronic phase of the infection [9]. The current hypothesis explaining these observations is that immediate treatment during AHI limits the formation of the viral reservoir [10,11] and preserves the immune system leading to better immune-mediated viral control [12]. Indeed, recent studies showed that in addition to a small viral reservoir, different components of the immune system, such as NK cells and CD8^+^ T-cell responses, can be attributed to HIV control in individual PTC cases [13,14].

Here, we describe a case of extraordinary PTC in a person who was diagnosed with acute HIV infection in 1998, received ART for 26 months and has been successfully controlling the virus for 23 years after cessation of ART. In this study, we performed a comprehensive viro-immunological analysis to delineate correlates of viral control.

## Materials and methods

### Study participants

In 1998, a 49-year-old man was diagnosed with AHI with symptoms of headache, fever, night sweats, a sore throat and lymphadenopathy, and included in a primary HIV infection study (ERA study). Two weeks after diagnosis he started a five-drug regimen (stavudine (40 mg twice daily), lamivudine (150 mg twice daily), abacavir (300 mg twice daily), nevirapine (400 mg once daily; 200 mg once daily during the first 2 weeks), and indinavir (1000 mg three times daily; after 2 months, he switched to indinavir 800 mg and ritonavir 100 mg twice daily) [15]. In March 2000, he switched to Combivir twice daily and efavirenz 600 mg once daily. At his own request, he discontinued treatment after 26 months of treatment.

**Fig. 1:**
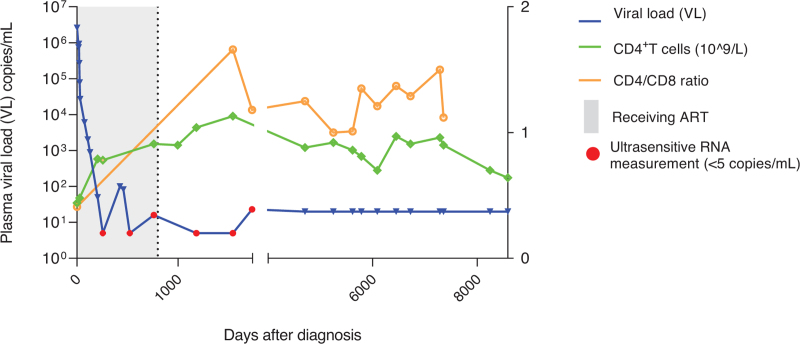
Clinical characteristics of the case.

Follow-up appointments continued after treatment interruption and included blood sampling for PBMC isolation and routine laboratory tests such as lymphocyte counts (CD4^+^ and CD4^+^/CD8^+^ ratio, using flowcytometry) and assessment of plasma HIV RNA load using diagnostic assays (Fig. 1). In addition, for study purposes, ultrasensitive plasma viral load PCRs were performed (detection threshold <5 copies/ml) [15].

Plasma viral load (left y-axis), CD4^+^ cell count, and CD4^+^/CD8^+^ ratio (right y-axis) were measured at every study visit. Detection limit of the standard diagnostic viral load assay was <400 copies/ml (1997–1999: day 0–365 after diagnosis), <40 copies/ml (1999–2021: day 365–8340 after diagnosis), and <20 (2021--present: 8340--present after diagnosis). Additional ultrasensitive viral load PCR tests (<5 copies/ml) were performed and are depicted as red dots.

The trial (ERA study; 96/232) was approved by the Medical Ethics Committee of the Amsterdam Medical Center. The person described in this study gave informed consent to participate in this trial, and continuation of blood sampling after the trial had ended.

Untreated HIV-infected individuals from the Amsterdam Cohort Studies on HIV and AIDS (ACS) and HIV-negative donors were included for comparison. For a detailed description, see Supplementary Methods and Supplementary Table 1.

### Genotyping

HLA and KIR genotyping was performed at the Department of Immunogenetics (Sanquin) by the PCR using sequence-based typing (SBT) method (GenDx Products, Utrecht, the Netherlands) and real-time (RT)-PCR (Thermofisher, West Hills, California, USA). The CCR5delta32 genotype was determined by PCR (Supplementary methods).

### Virological analysis

Proviral DNA load was determined by qPCR on the Lightcycler 480 using the GoTaq qPCR Master Mix (Promega, Madison, Wisconsin, USA) using a primer set detecting a conserved region in the HIV *pol* gene. HIV intact proviral DNA was quantified using a multiplex ddPCR assay, targeting both the Ψ (*psi*) region and part of the envelope glycoprotein (*env*) region.

The quantitative viral outgrowth assay using CD8^+^ T-cell depleted patient PBMC was performed to assess the level of infectious virus and to isolate replication-competent virus.

HIV replication kinetics were determined by inoculation of CD8^+^-depleted PBMC from healthy donors using 40 TCID50 per 1 × 10^6^ cells. Every fourth day, freshly stimulated CD8^+^-depleted PBMC were added. Viral replication was measured regularly using an in-house p24 ELISA, with a detection limit of 20 ng/ml.

HIV co-receptor use was determined by geno2pheno online tool and confirmed by an infection assay using human glioblastoma (U87) cell lines stably expressing CD4^+^ and the HIV coreceptors CCR5 or CXCR4.

The NL4.3 Ba-L P255A mutant was constructed using site-directed mutagenesis and the replication rate was determined on PBMC from healthy donors using 80 TCID50 per 1 x 10^6^ cells.

For detailed methods, see supplementary methods.

### Immunological analysis

PBMCs were used for immune phenotyping (activation, exhaustion and senescence) by flow cytometry. T-cell functionality was determined by the analysis of intracellular cytokine production and proliferative capacity upon stimulation using overlapping peptide pools (HIV: Gag and Nef; CMV: pp65). For detailed methods, see Supplementary Methods.

### HIV-specific antibody analysis

HIV-specific serum IgG titers were determined by Luminex assay using three well characterized stabilized, native-like Env trimers of (BG505 SOSIP, ConM SOSIP, and AMC011 SOSIP) and predominant epitopes of Clade B HIV Env (JRCSF) (gp41 monomer, gp120 monomer, and the V3 loop peptide on a scaffold). Neutralization assays were performed using the pseudoviruses of the Global Panel of HIV Env Reference Clones (NIH AIDS Reagent Program). For detailed methods, see supplementary methods.

### Antiretroviral drug screening

Quantitative testing of 11 frequently used antiretroviral drugs (amprenavir, bictegravir, darunavir, dolutegravir, doravirine, efavirenz, etravirine, lopinavir, nevirapine, raltegravir, and rilpivirine, and nevirapine) was performed by means of a validated LC-MS/MS method by the Clinical Pharmacology Department of the University Medical Center in Utrecht, the Netherlands.

## Results

At presentation, the person had an HIV viral load of 2.7 million copies/ml plasma, a p24-antibody positive ELISA test and an indeterminate western blot (Fiebig stage 4). The viral load declined to 27 000 copies/ml 2 weeks after ART initiation and became undetectable after 7 months of ART. Except for a viral blip at 15 months after ART start (82 copies/ml), the plasma viral load remained undetectable. In October 2000, the person discontinued ART. After treatment interruption, the plasma viral load remained undetectable for 23 years using standard diagnostic assays, with the exception of a single viral blip 7 months after treatment interruption (400 copies/ml). However, plasma viral load was detectable at very low levels using an ultrasensitive PCR (<5 copies/ml) (Fig. 1, blue dots). Furthermore, no clinical signs of any HIV-associated disease were noticed during follow-up. Serum levels of eleven frequently used antiretroviral drugs were undetectable at 18 years after treatment interruption.

At the time of diagnosis, CD4^+^ T-cell count was 0.44 × 10^9^ cells/l, which recovered to levels above 0.7 × 10^9^ CD4^+^ T cells/l after start of ART and remained stable up to 23 years after treatment interruption. Immune phenotyping of PBMC performed 18 years after treatment interruption showed low levels of CD4^+^ and CD8^+^ T-cell activation, exhaustion and senescence as compared to untreated people with HIV (characteristics displayed in Supplementary Table 1), and more comparable to levels observed in HIV-negative donors (Supplementary Figure 1).

The person was HLA-A∗02, A∗68 and homozygous for B∗44 : 02 and did not harbor the protective HLA alleles HLA-B∗57 or HLA-B∗27. KIR genotyping showed that this person is KIR2DL1, KIR2DL3, KIR2DL4, KIR3DL1, KIR3DL2, KIR3DL3, KIR2DS4, KIR2DP1, and KIR3DP1 positive. Moreover, the person carried the homozygous wild-type genotype for CCR5. Taken together, in addition to the high plasma viral load at diagnosis, the HLA genotype, with lack of protective HLA alleles, indicates that the case does not have the profile of an elite controller.

**Fig. 2:**
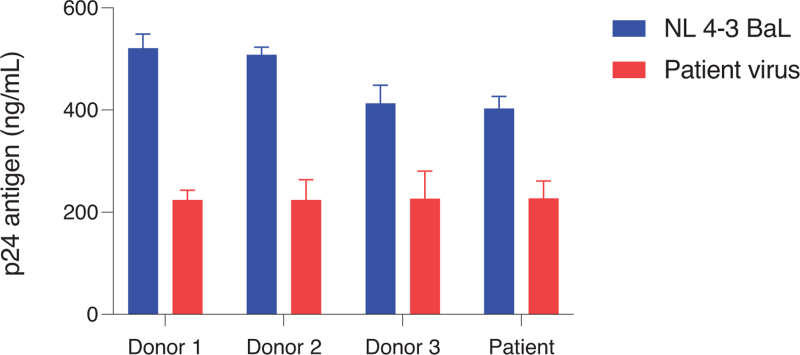
HIV infectivity assay *in vitro*.

At 18 years after treatment interruption, a cellular proviral DNA load of 1500 *pol*-DNA copies/10^6^ CD4^+^ T cells and 37 intact proviruses/10^6^ CD4^+^ T cells was detected. Quantitative viral outgrowth assays using CD4^+^ T cells resulted in isolation of replicating HIV (48 IU/10^6^ CD4^+^ T cells) at 1 month after diagnosis, whereas no replicating HIV could be isolated 15 months after start ART (during the first viral blip) and 18 years after treatment interruption (<0.03 IU/10^6^ CD4^+^ T cells). Full length viral sequencing of the replication competent virus (1 month after diagnosis) and proviral DNA (18 years after treatment interruption) showed that the virus was a clade B variant with only a limited number of mutations and no major insertions or deletions when compared with HXB2 consensus. Over time (1 month after infection versus 18 years after treatment interruption), no amino acid changes in Gag, three amino acid changes in Pol, and four amino acid changes in Env, with no changes in the V3 region, were observed. On the basis of the envelope sequence, the virus used the CCR5 co-receptor for viral entry. CCR5 co-receptor use of the replication-competent HIV variant was confirmed using U87 cells stably expressing CD4^+^ and the co-receptors CCR5 and CXCR4. The replication-competent HIV variant was able to replicate equally well in both CD4^+^ T cells from HIV-negative donors and from the case (Fig. 2). Moreover, CD4^+^ T cells from the case were equally susceptible to infection with the laboratory strain NL4-3 BaL compared with CD4^+^ T cells from HIV-negative donors (Fig. 2). This suggests that a replication-competent virus was present early after infection, and that CD4^+^ T cells from the case supported HIV replication indicative of the absence of any intrinsic viral restriction factors.

The HIV variant obtained from the person 1 month after diagnosis (red) was used to infect PBMC obtained from healthy donors (*n* = 3) and from the person. The NL 4-3 BaL lab strain (blue) was used as a positive control.

**Fig. 3:**
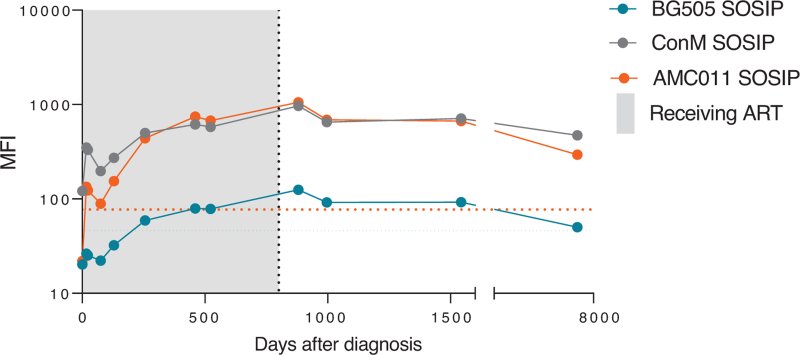
HIV envelope binding IgG in serum.

Next, the functionality of the humoral and cellular HIV-specific immune response was characterized during follow-up (1998–2022). The HIV-Env IgG antibody titers binding Clade B AMC011 SOSIP and consensus group M (ConM) SOSIP, as well as Env subdomains increased rapidly after diagnosis and remained stable during follow-up (Fig. 3 and Supplementary Figure 2). However, no neutralization antibodies against a global virus panel could be detected in serum 18 years after treatment interruption (Supplementary Table 2). The persisting antibody response is suggestive of ongoing exposure to the virus. Also, a western blot was performed 18 years after treatment interruption, which showed broad antibody responses to Gag, Pol, and Env, indicating antibody responses targeting all viral proteins.

IgG antibody responses against several Env trimers [BG505 SOSIP (clade A); ConM SOSIP (consensus group M); AMC011 SOSIP (clade B)] were measured. A serum dilution of 1 : 1000 was used for this assay. The dotted lines represent the SOSIP specific cut-off, reflecting the IgG binding response of a pool of HIV negative serum. MFI, median fluorescence intensity.

**Fig. 4:**
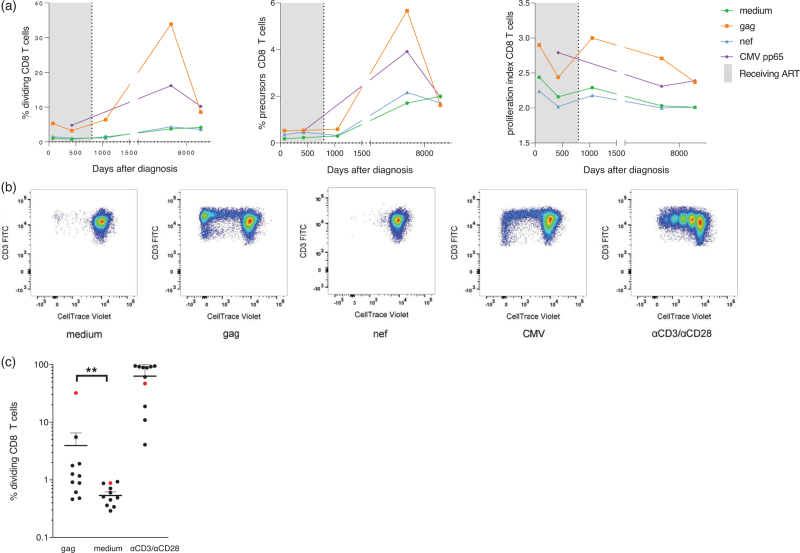
HIV-specific CD8^+^ T-cell response.

Polyfunctionality of HIV-Gag specific CD4^+^ and CD8^+^ T cells as determined by intracellular cytokine production (IL-2, IFN-γ, TNF-α, MIP-1β) and degranulation (CD107α) 18 years after TI was comparable to untreated people with HIV (Supplementary Figure 4). However, a strong proliferative CD8^+^ T-cell response against HIV Gag, but not Nef, with a high precursor frequency and proliferation index was observed over time (Fig. 4). The proliferative capacity of HIV Gag-specific CD8^+^ T cells of the patient was higher as compared to untreated people with HIV (characteristics displayed in Supplementary Table 1). A strong proliferative response was also observed for CD4^+^ T cells; however, antigen specificity could not be determined due to high background responses (Supplementary Figure 3). The CD4^+^ T-cell response to HIV Gag was comparable to that of CD4^+^ T cells from untreated people with HIV (characteristics displayed in Supplementary Table 1).

(a) Longitudinal frequencies of dividing CD8^+^ T cells, precursor CD8^+^ T cells, and proliferation indices upon peptide pool stimulation. (b) Representative flowcytometry plots (y-axis CD3 FITC and x-axis CellTrace Violet). (c) Percentage of dividing CD8^+^ T cells upon Gag peptide stimulation, compared to medium (negative control) and α-CD3/α-CD28 stimulation (positive control). Red dot: person described in this study. Black dots: untreated, chronic HIV positive individuals from the ACS cohort (Supplementary Methods).

**Fig. 5:**
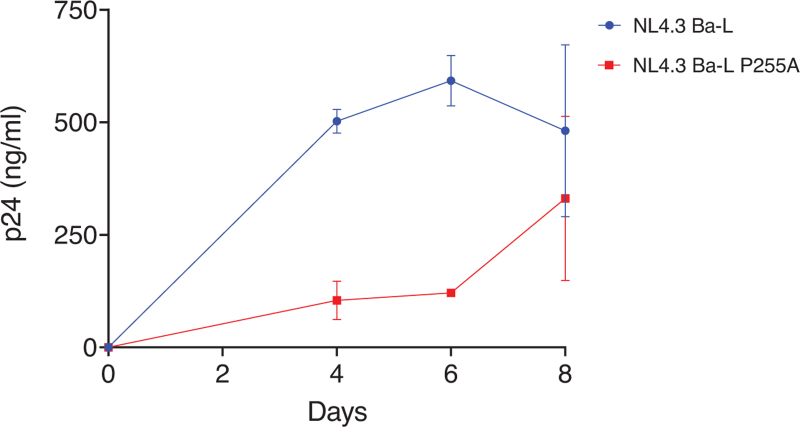
Viral replication rate of NL4.3 Ba-L P255A.

As the HLA-B∗44 : 02 has previously been associated with high HIV-specific CD8^+^ T-cell responses and prolonged HIV control [16–18], we next determined whether the strong Gag-specific CD8^+^ T-cell response was associated with viral escape mutations in Gag. We observed a loss of the B∗44 : 02 restricted predicted IL10 epitope (IEVKDTKEAL) due to a mutation at the anchor residue (INVKDTKEAL). Moreover, a mutation at position 7 (P255A) in the previously identified HLA-B∗44 : 02 restricted Gag epitope (WMTNNPPIPVGDIYKRW) [18] was observed, which may result in loss of recognition by the T-cell receptor (TCR). To determine whether the P255A mutation in the HIV capsid protein had an effect on viral replication, it was cloned into the NL4.3 Ba-L molecular clone and analyzed for replication fitness in PBMC. We observed a slow replication of the NL4.3 Ba-L P255A variant as compared to the wild-type variant, during the first 5 days of the experiment (Fig. 5). These data suggest that a strong and durable response of Gag-specific CD8^+^ T cells in combination with slower replication associated with the P255A mutation is related to long-term viral control in our case.

Viral replication rate of NL4.3 Ba-L (blue) and NL4.3 Ba-L P255A (red) in PBMC from healthy donors determined by p24 production in the culture supernatant over 8 days.

## Discussion

Here, we describe a person who controlled HIV for 23 years after TI. The high viral load in plasma during AHI as well as the absence of the protective HLA-B∗57 or B∗27 alleles confirm that this person does not have the profile of an elite controller. The isolation of a fully replication-competent HIV shortly after ART initiation and in-vitro susceptibility of CD4^+^ T cells of the person to HIV infection indicated that there were no intrinsic viral restriction factors present. Moreover, at 18 years after treatment interruption, the level of intact proviral DNA was comparable to levels observed in chronically infected individuals [19]. Interestingly, the strong HIV-Gag proliferative CD8^+^ T-cell response was already present two months after infection and was maintained during the entire follow-up. These data suggest that the high numbers of precursor CD8^+^ T cells recognizing the conserved HIV-Gag protein and their robust proliferative response upon antigen exposure are important factors in the maintenance of PTC.

HIV immune control by T-cell responses has been extensively described in elite controllers carrying protective HLA-alleles [20,21]. PTCs were shown to have in general low levels of CD8^+^ T-cell responses [6,22]; however, a CD8^+^ T-cell mediated controlling immune response in a PTC has been described [13]. In that case, a robust polyfunctional Gag-specific CD8^+^ T-cell response was elicited already before ART initiation, and residual viral replication, as evidenced by viral blips after treatment interruption, was considered an important driver of the high antiviral response. Moreover, the emergence of escape mutations in HLA-restricted CD8^+^ T-cell epitopes may have led to loss of immune control in that case [13]. The person described was homozygous for the HLA Bw4 allele B∗44 : 02. This allele has previously been associated with a lower peak viral load, a high HIV-specific CD8^+^ T-cell response and prolonged HIV control during untreated infection [16–18]. The high viral load that we observed in this person early after infection contradicts that the HLA-B∗44 allele accounts for control in the case described here. However, we did observe a mutation (P255A) in a previously identified HLA-B∗44 : 02 restricted Gag-epitope and this mutation was associated with decreased replication in vitro.

The HLA Bw4 allotype and Bw4 ligand KIR alleles homozygosity have also been associated with HIV control [17,23], a lower Gag-specific T-cell response [18], and higher natural killer (NK) cell activity [23]. In a recently published PTC case report, a favorable genetic background (Bw4 and KIR) may have contributed to NK-cell mediated viral control [14]. In that case, a high frequency of NKG2C CD57 memory-like NK cells and CD8^+^ γδ T cells expressing NKG2C with increased cytotoxicity for HIV infected cells were found [14]. In contrast, the case we describe had a robust Gag-specific CD8+ T-cell response, did not carry protective KIR alleles, and no increased in-vivo NK cell activation (data not shown) was observed.

Antibody responses targeting viral Env were stable during complete follow-up, but did not display any neutralizing activity. Broadly neutralizing antibodies (bNAbs) can control HIV replication upon treatment interruption [24–26]; however, these need to be very potent and broad, be present at high concentrations and in a combination of at least three bNAbs to prevent viral escape and loss of control. Therefore, it is very unlikely that the observed nonneutralizing antibody response contributes to viral control in our case.

Taken together, our findings show that in the case described here a potent CD8^+^ T-cell proliferative response in combination with slower viral replication associated with the P255A Gag-mutation accounts for 23 years of PTC.

A small viral reservoir due to early treatment initiation appears to be one of several prerequisites for PTC, confirming the importance of early diagnosis of HIV infection and starting ART during AHI. Nevertheless, modeling of the viral reservoir dynamics has shown that a large reduction of the viral reservoir is needed to delay viral rebound only by a few weeks [27], which indicates that additional induction of the HIV-specific immune responses is necessary to achieve PTC. This PTC case, together with earlier case reports, shows that different components of the immune system, for example, NK cells and CD8^+^ T-cell responses, individually contribute to PTC [13,14].

## Acknowledgements

The authors would like to thank the study participant for enabling us to report his case and for his long-term commitment. The ERA study (1998) was financially supported by a private foundation, which did not wish to be named. This study was financially supported by the Dutch Aidsfonds (P-54901).

J.P., G.J.d.B., and N.K. designed the study. M.N. and J.S. contributed to study conceptualization. P.v.P, L.v.P, K.v.d.S, N.B., M.G., A.v.N., K.v.D., B.B.N., M.v.d.E., J.B., Mv.L., S.J., and W.S. performed the experiments and/or collected data. R.S. provided resources for an experiment. P.v.P, N.K., G.J.d.B., and J.P. did the formal analysis and project administration. P.v.P, and N.K. wrote the original draft. P.v.P, N.K., L.v.P., and K.v.d.S., contributed to visualization. J.P., G.J.d.B., and N.K. were responsible for funding acquisition and supervision. M.K. contributed to supervision. All authors reviewed and approved the final manuscript. All authors had full access to all the data and had final responsibility for the decision to submit for publication.

All data from this study is available upon request to P.v.P. (p.vanpaassen@amsterdamumc.nl).

### Conflicts of interest

G.J.d.B. received consulting fees (Gilead Sciences, Takeda, Exevir) and research grants (Gilead Sciences, ViiV, MacAidsfund) outside of the submitted work and paid to her institution. All other authors declare no competing interests. This study was financially supported by the Dutch Aidsfonds (P-54901). The funders of the study had no role in study design, data collection, data analysis, data interpretation, writing this case report, or decision to submit for publication.

## Supplementary Material

Supplemental Digital Content

## Supplementary Material

Supplemental Digital Content

## Supplementary Material

Supplemental Digital Content

## References

[1] Chun TW Davey RT Jr Engel D Lane HC Fauci AS. Re-emergence of HIV after stopping therapy. *Nature* 1999; 401:874–875. 10553903 10.1038/44755

[2] Hütter G Nowak D Mossner M Ganepola S Müßig A Allers K. Long-term control of HIV by CCR5 Delta32/Delta32 stem-cell transplantation. *N Engl J Med* 2009; 360:692–698. 19213682 10.1056/NEJMoa0802905

[3] Gupta RK Abdul-Jawad S McCoy LE Mok HP Peppa D Salgado M. HIV-1 remission following CCR5Δ32/Δ32 haematopoietic stem-cell transplantation. *Nature* 2019; 568:244–248. 30836379 10.1038/s41586-019-1027-4 PMC7275870

[4] Jensen BO Knops E Cords L Lübke N Salgado M Busman-Sahay K. In-depth virological and immunological characterization of HIV-1 cure after CCR5Δ32/Δ32 allogeneic hematopoietic stem cell transplantation. *Nat Med* 2023; 29:583–587. 36807684 10.1038/s41591-023-02213-x PMC10033413

[5] Aribi A Budde LE Alvarnas JC Smith EP Salhotra A Al Malki MM. Durable leukemia and HIV remission without antiviral therapy following an allogeneic hematopoietic stem cell transplantation (alloHCT) using a donor with CCR5-Δ32/Δ32 homozygosity for an acute myeloid leukemia (AML) patient. *Blood* 2022; 140: (Suppl 1): 7597–7598.

[6] Sáez-Cirión A Bacchus C Hocqueloux L Avettand-Fenoel V Girault I Lecuroux C. Posttreatment HIV-1 controllers with a long-term virological remission after the interruption of early initiated antiretroviral therapy ANRS VISCONTI Study. *PLoS Pathog* 2013; 9:e1003211. 23516360 10.1371/journal.ppat.1003211 PMC3597518

[7] Persaud D Gay H Ziemniak C Chen YH Piatak M Chun T-W. Absence of detectable HIV-1 viremia after treatment cessation in an infant. *N Engl J Med* 2013; 369:1828–1835. 24152233 10.1056/NEJMoa1302976 PMC3954754

[8] Frange P Faye A Avettand-Fenoël V Bellaton E Descamps D Angin M. HIV-1 virological remission lasting more than 12 years after interruption of early antiretroviral therapy in a perinatally infected teenager enrolled in the French ANRS EPF-CO10 paediatric cohort: a case report. *Lancet HIV* 2016; 3:e49–e54. 26762993 10.1016/S2352-3018(15)00232-5

[9] Namazi G Fajnzylber JM Aga E Bosch RJ Acosta EP Sharaf R. The Control of HIV After Antiretroviral Medication Pause (CHAMP) Study: posttreatment controllers identified from 14 clinical studies. *J Infect Dis* 2018; 218:1954–1963. 30085241 10.1093/infdis/jiy479 PMC6217727

[10] Chun T-W Justement JS Moir S Hallahan CW Maenza J Mullins JI. Decay of the HIV reservoir in patients receiving antiretroviral therapy for extended periods: implications for eradication of virus. *J Infect Dis* 2007; 195:1762–1764. 17492591 10.1086/518250

[11] Ananworanich J Schuetz A Vandergeeten C Sereti I de Souza M Rerknimitr R. Impact of multi-targeted antiretroviral treatment on gut T cell depletion and HIV reservoir seeding during acute HIV infection. *PLoS One* 2012; 7:e33948. 22479485 10.1371/journal.pone.0033948 PMC3316511

[12] Le T Wright EJ Smith DM He W Catano G Okulicz JF. Enhanced CD4+ T-cell recovery with earlier HIV-1 antiretroviral therapy. *N Engl J Med* 2013; 368:218–230. 23323898 10.1056/NEJMoa1110187 PMC3657555

[13] Blazkova J Gao F Marichannegowda MH Justement JS Shi V Whitehead EJ. Distinct mechanisms of long-term virologic control in two HIV-infected individuals after treatment interruption of antiretroviral therapy. *Nat Med* 2021; 27:1893–1898. 34711975 10.1038/s41591-021-01503-6

[14] Climent N Ambrosioni J González T Xufré C Casadellà M Noguera-Julian M. Immunological and virological findings in a patient with exceptional posttreatment control: a case report. *Lancet HIV* 2023; 10:e42–e51. 36354046 10.1016/S2352-3018(22)00302-2

[15] Weverling GJ Lange JM Jurriaans S Prins JM Lukashov VV Notermans DW. Alternative multidrug regimen provides improved suppression of HIV-1 replication over triple therapy. *AIDS* 1998; 12:F117–F122. 9708401 10.1097/00002030-199811000-00003

[16] Tang J Cormier E Gilmour J Price MA Prentice HA Song W. Human leukocyte antigen variants B∗44 and B∗57 are consistently favorable during two distinct phases of primary HIV-1 infection in Sub-Saharan Africans with several viral subtypes. *J Virol* 2011; 85:8894–8902. 21715491 10.1128/JVI.00439-11 PMC3165830

[17] Flores-Villanueva PO Yunis EJ Delgado JC Vittinghoff E Buchbinder S Leung JY. Control of HIV-1 viremia and protection from AIDS are associated with HLA-Bw4 homozygosity. *Proc Natl Acad Sci U S A* 2001; 98:5140–5145. 11309482 10.1073/pnas.071548198 PMC33177

[18] Zhang X Huang X Xia W Li W Zhang T Wu H. HLA-B∗44 is associated with a lower viral set point and slow CD4 decline in a cohort of Chinese homosexual men acutely infected with HIV-1. *Clin Vaccine Immunol* 2013; 20:1048–1054. 23677320 10.1128/CVI.00015-13 PMC3697455

[19] Kwaa AK Garliss CC Ritter KD Laird GM Blankson JN. Elite suppressors have low frequencies of intact HIV-1 proviral DNA. *AIDS* 2020; 34:641–643. 31895150 10.1097/QAD.0000000000002474 PMC7610219

[20] Migueles SA Laborico AC Shupert WL Sabbaghian MS Rabin R Hallahan CW. HIV-specific CD8+ T cell proliferation is coupled to perforin expression and is maintained in nonprogressors. *Nat Immunol* 2002; 3:1061–1068. 12368910 10.1038/ni845

[21] Sáez-Cirión A Lacabaratz C Lambotte O Versmisse P Urrutia A Boufassa F. HIV controllers exhibit potent CD8 T cell capacity to suppress HIV infection ex vivo and peculiar cytotoxic T lymphocyte activation phenotype. *Proc Natl Acad Sci U S A* 2007; 104:6776–6781. 17428922 10.1073/pnas.0611244104 PMC1851664

[22] Collins DR Gaiha GD Walker BD. CD8(+) T cells in HIV control, cure and prevention. *Nat Rev Immunol* 2020; 20:471–482. 32051540 10.1038/s41577-020-0274-9 PMC7222980

[23] Maruthamuthu S Rajalingam R Pandian K Madasamy S Manoharan M Pitchai L. Inhibitory natural killer cell receptor KIR3DL1 with its ligand Bw4 constraints HIV-1 disease among South Indians. *AIDS* 2018; 32:2679–2688. 30289808 10.1097/QAD.0000000000002028

[24] Gaebler C Nogueira L Stoffel E Oliveira TY Breton G Millard KG. Prolonged viral suppression with anti-HIV-1 antibody therapy. *Nature* 2022; 606:368–374. 35418681 10.1038/s41586-022-04597-1 PMC9177424

[25] Gunst JD Pahus MH Rosás-Umbert M Lu IN Benfield T Nielsen H. Early intervention with 3BNC117 and romidepsin at antiretroviral treatment initiation in people with HIV-1: a phase 1b/2a, randomized trial. *Nat Med* 2022; 28:2424–2435. 36253609 10.1038/s41591-022-02023-7 PMC10189540

[26] Niessl J Baxter AE Mendoza P Jankovic M Cohen YZ Butler AL. Combination anti-HIV-1 antibody therapy is associated with increased virus-specific T cell immunity. *Nat Med* 2020; 26:222–227. 32015556 10.1038/s41591-019-0747-1 PMC7018622

[27] Hill AL Rosenbloom DIS Fu F Nowak MA Siliciano RF. Predicting the outcomes of treatment to eradicate the latent reservoir for HIV-1. *Proc Natl Acad Sci U S A* 2014; 111:13475–13480. 25097264 10.1073/pnas.1406663111 PMC4169952

