# Supplementary material for: Virological and immunological correlates of HIV posttreatment control after temporal antiretroviral therapy during acute HIV infection
Source: AIDS. 2023 Sep 11;37(15):2297–304. doi: 10.1097/QAD.0000000000003722 (PMC10653294; doi:10.1097/QAD.0000000000003722)
Supplement: Supplemental Digital Content [file aids-37-2297-s001.pdf]

Supplementary Figures

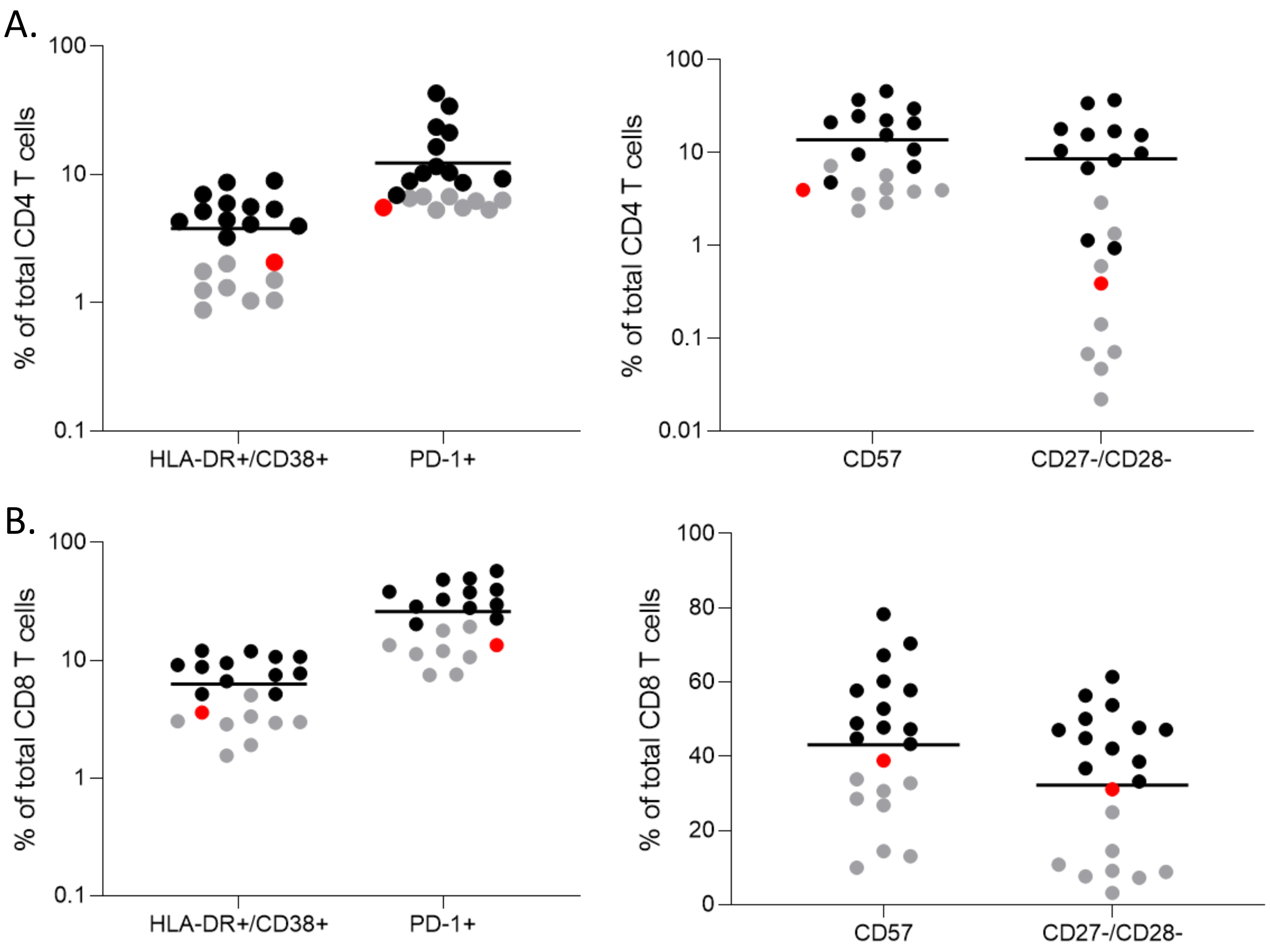

Supplementary Figure 1. Activation, exhaustion and senescence of CD4 (A) and CD8 (B) T cells.

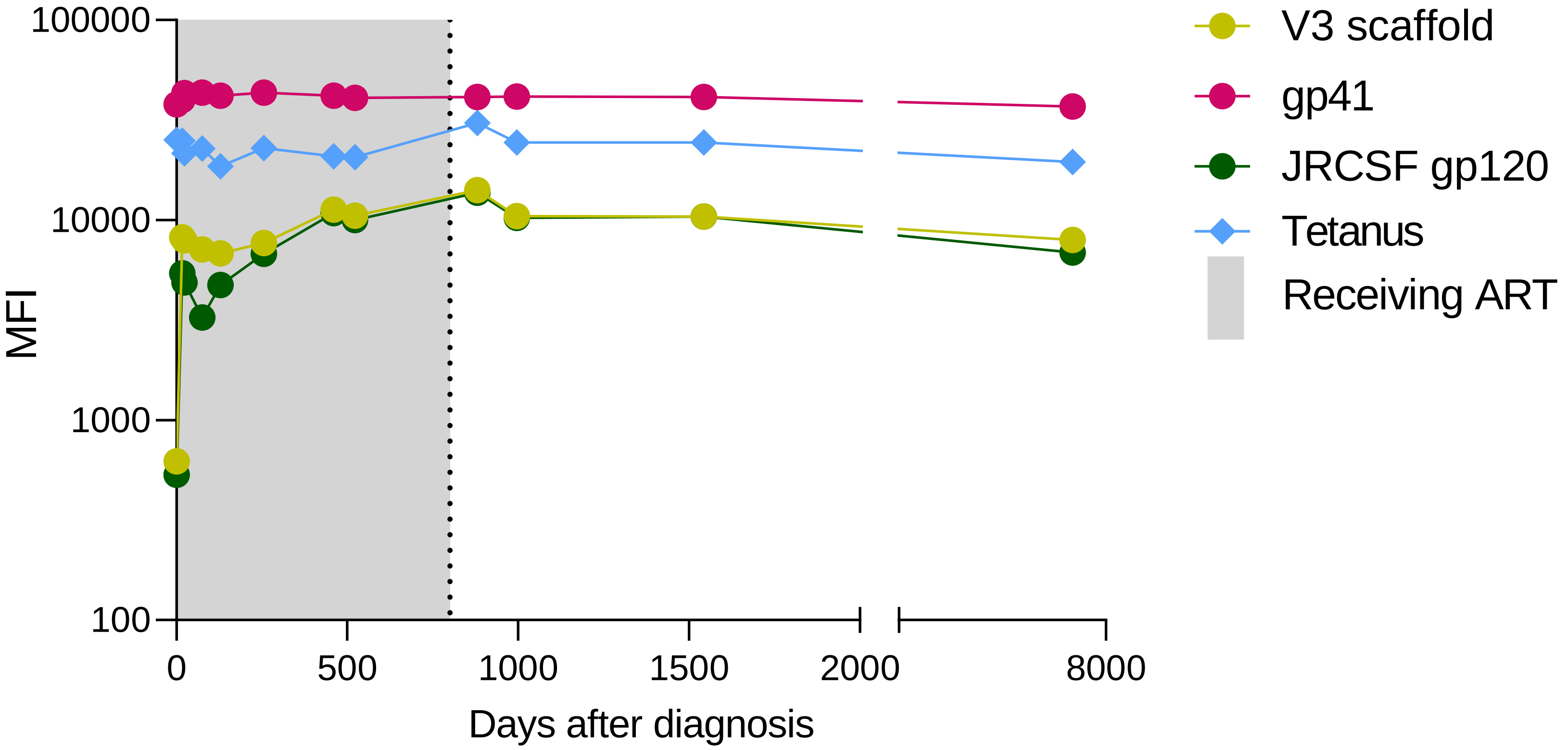

Supplementary Figure 2. IgG antibody responses

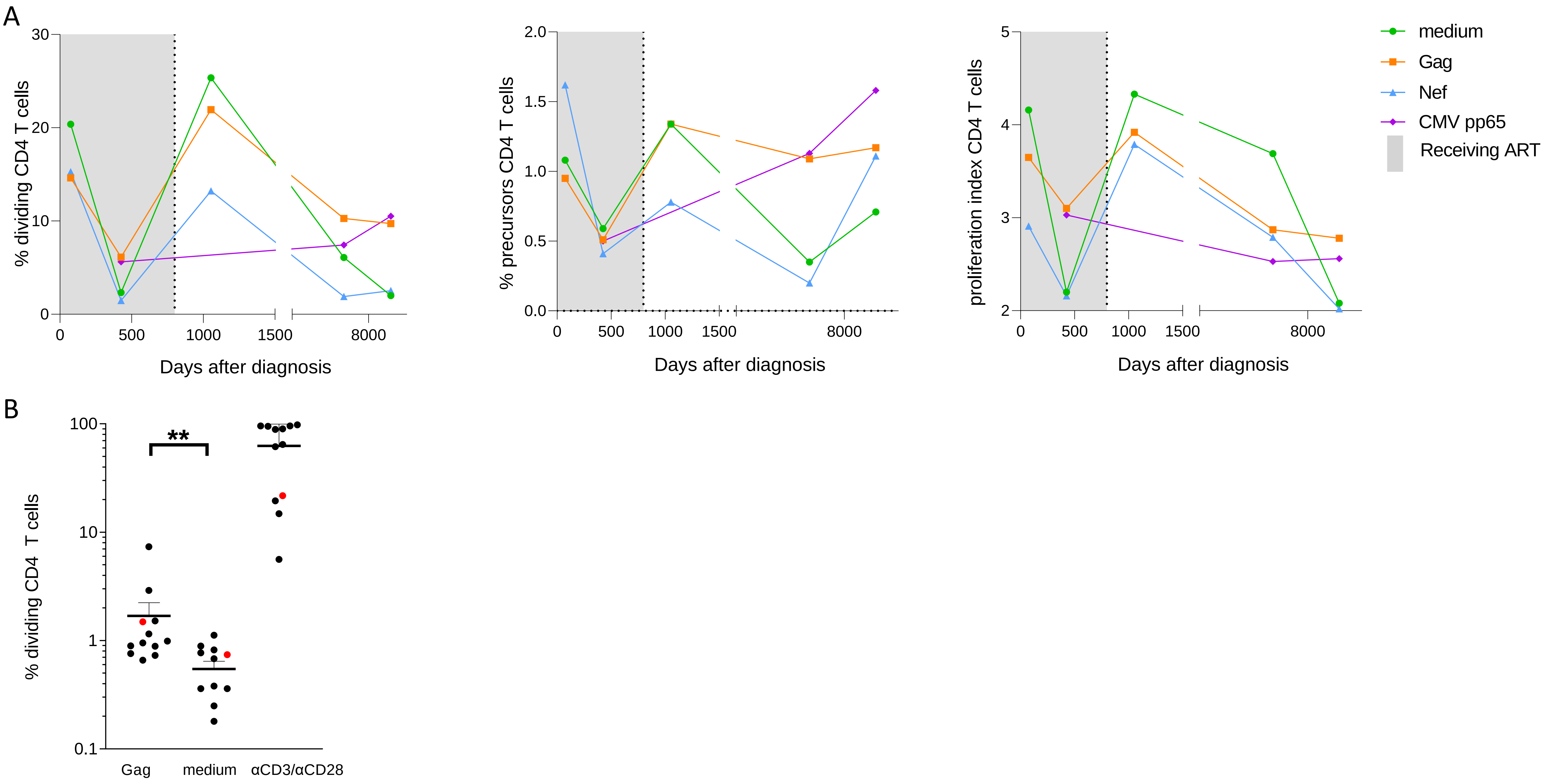

Supplementary Figure 3. HIV specific CD4 T cell response.

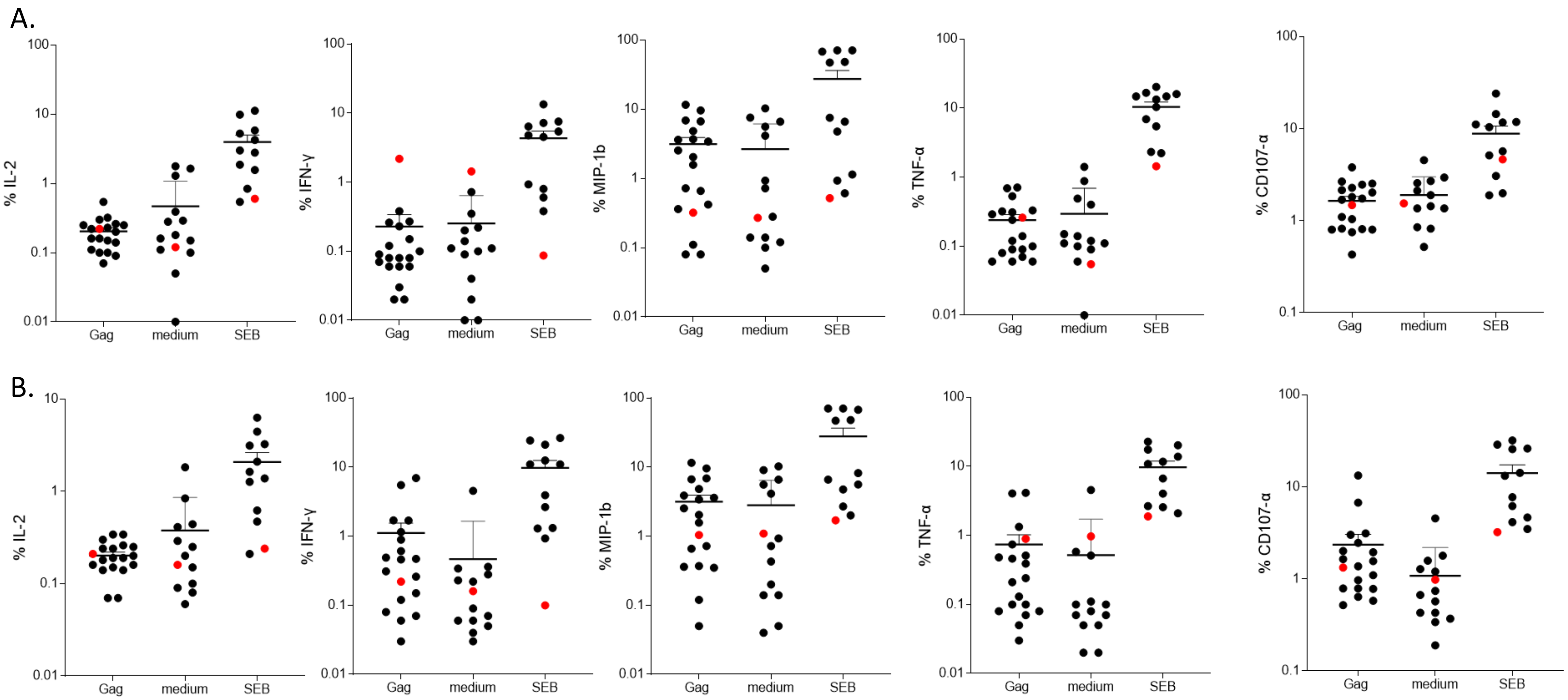

Supplementary Figure 4. T cell functionality
