# Supplementary material for: Virological and immunological correlates of HIV posttreatment control after temporal antiretroviral therapy during acute HIV infection
Source: AIDS. 2023 Sep 11;37(15):2297–304. doi: 10.1097/QAD.0000000000003722 (PMC10653294; doi:10.1097/QAD.0000000000003722)
Supplement: Supplemental Digital Content [file aids-37-2297-s002.docx]

**Supplementary Methods**

*Patient and donor material*

PBMC from blood donors from the Dutch national blood bank in Amsterdam, the Netherlands ([www.sanquin.nl](http://www.sanquin.nl)), and untreated chronic HIV infected participants from the Amsterdam Cohort Studies on HIV infection and AIDS (ACS) were included in the analysis for comparison. Characteristics of untreated chronic HIV infected participants are displayed in supplementary table 1.

The ACS has been conducted in accordance with the ethical principles set out in the declaration of Helsinki and was approved by the institutional review board of the Academic Medical Center (AMC). The use of materials from blood donors has been approved by the Ethics Advisory Body of the Sanquin Blood Supply Foundation in Amsterdam. Written informed consent was obtained from all participants.

**Virological analysis**

*Analysis of proviral DNA*

CD4+ T cells were isolated from PBMC using MACS Microbeads (Miltenyi Biotec, Bergisch Gladbach, Germany), subsequently genomic DNA was isolated using the DNeasy Blood and tissue kit (Qiagen), according to manufacturer’s guidelines. The proviral DNA load was determined by qPCR on the Lightcycler 480 using the GoTaq qPCR Master Mix (Promega, Madison, WI, USA). Primers sets used were HIV-pol-B (Fw) 5’- TAACCTGCCACCTGTAGTAGCAAAAGAAAT-3' and Pol-E (rev) 5’-ATGTGTACAATCTAGTTGCCA-3' detecting a conserved region in the HIV *pol* gene. HIV intact proviral DNA was quantified using a multiplex ddPCR assay, targeting both the Ψ (*psi*) region and part of the *env* region [1]. The DNA shearing index (DSI) is a measure used to correct for the DNA shearing that may have occurred during the experimental procedure. To calculate the DSI, two regions of the RPP30 cellular genes are quantified in parallel. These two regions are specifically chosen because they are at equal distance as the *psi* and *env* regions of the IPDA. The DSI is calculated by dividing the mean defective copies by the mean defective + intact copies. As a positive control Jlat 15.4 cells (NIH AIDS Reagent Program, 9848) were used. DNA isolated from PBMCs of HIV-negative donors was used as a DNA template control and water as a no template control. Cycle conditions were performed according to manufacturer’s protocol, except for the multiplex ddPCR where an annealing temperature of 55°C was used. Analysis was performed using Quantasaft version 1.7.4.

*Viral sequencing*

Full length viral sequences were generated by Sanger sequencing of clonal virus isolates obtained in viral outgrowth assays or through single proviral genome amplification using DNA obtained from CD4+ T cells of the patient. Sequencing was performed using the ABI prism Big Dye Terminaters v1.1 cyclesequencing kit (Applied Biosystems, Foster City, CA, USA). Data was analyzed using BioEdit (v 7.0.5)[2].

*Quantitative Viral Outgrowth Assay*

Isolation of replication-competent virus was performed using CD8+ T cell depleted patient PBMC as described previously [3]. Patient PBMCs were depleted for CD8+ T cells using MACS Microbeads (Miltenyi Biotec, Bergisch Gladbach, Germany) and prestimulated for 48 hours using Iscove’s modified Dulbecco’s medium (IMDM) supplemented with 10% (v/v) heat inactivated fetal calf serum (FCS), penicillin (100 U/ml), streptomycin (100 μg/ml), phytohemagglutinin (PHA; Remel Europe, Dartford, England, UK; 1 µg/ml) and maintained in a humidified 10% CO2 incubator at 37 °C. Subsequently, the cells were co-cultured with 2-day PHA-stimulated donor PBMC in IMDM supplemented with 10% (v/v) heat inactivated FCS, penicillin (100 U/ml), streptomycin (100 μg/ml) and IL-2 (20 U/ml; Chiron Benelux). Culture supernatants were regularly analyzed for viral replication using an in-house p24 antigen enzyme-linked immunosorbent assay (ELISA) [3]. Every 7-days, fresh PHA-stimulated donor PBMC were added to propagate the culture.

*In Vitro HIV replication assay*

CD8+-depleted PBMC from HIV-negative donors or the case were cultured in IMDM supplemented with 10% (v/v) heat inactivated FCS, penicillin (100 U/ml), streptomycin (100 μg/ml) and stimulated with anti-CD3 /anti-CD28 (2ug/ml) for 48 hours. After washing, the cells were resuspended in IMDM supplemented with 10% (v/v) heat inactivated FCS, penicillin (100 U/ml), streptomycin (100 μg/ml) and IL-2 (20 U/ml; Chiron Benelux) at a cell density of 1x10^6^ cells/ml. Subsequently, the cells were inoculated with HIV at a TCID50 of 40 per 1x10^6^ cells. Every 4^th^ day, freshly stimulated CD8+-depleted PBMC were added. Viral replication was measured regularly using an in-house p24 ELISA[3].

*Determination of Co-receptor use*

HIV coreceptor use was determined by geno2pheno online tool [4] and by infection of human glioblastoma (U87) cell lines stably expressing CD4+ and wild-type CCR5 (CCR5 cell line), CXCR4 (CXCR4 cell line), chimeric CCR5-CXCR4 coreceptors (Fc-2, Fc-4b, Fc-5, Fc-6, Fc-7 cell lines) or no coreceptor (SHAM cell line)[5]. Cells were cultured according to a protocol described previously and seeded into flat bottomed 96-well tissue culture plates (4000 cells/well) in 100 μl of culturing media, incubated at 37 °C with 5% CO_2_. Cells were inoculated with HIV (4 TCID50/well, in triplicate). Virus production in supernatant was measured on day 7, 10, 14 and 21 using an in-house p24 antigen ELISA.

*Construction and replication fitness of NL4.3 Ba-L P255A*

For the construction of NL4.3 Ba-L P255A, the *gag* region of the molecular clone NL4-3.Ba-L was removed using restriction enzymes BssHII and ApaI and then cloned into the pGEM T easy vector (Promega, Madison, Wisconsin, USA). The NL4-3.Ba-L molecular clone contains a glycine at position 248 instead of an alanine, and therefore the P255A was introduced in combination with the G248A mutation in the *gag* region using site directed mutagenesis as described by the manufacturer (Quick exchange kit, Stratagene). The gag insert was sequenced to confirm successful mutagenesis using primers T7 (5′-TAATACGACTCACTATAGGG-3′) and SP6 (5′-GATTTAGGTGACACTATAG-3′). The mutated *gag* insert was ligated into the full length molecular NL4-3.Ba-L clone using BssHII and ApaI restriction sites and the introduction of mutations was confirmed by sequence analysis. The full-length NL4-3.Ba-L molecular clones were then transfected into 293T cells using the calcium phosphate method [6]. Virus production from the 293T cells was analyzed at day 3 and day 7 after transfection using an in house p24 ELISA[3]. Virus titers were determined by 50% tissue culture infectious dose (TCID50) as previously described[3]. Viral replication rates of the NL4.3 Ba-L and NL4.3 Ba-L P255A were determined in PBMC from healthy donors. In brief, PBMC were stimulated for two days PHA (1 µg/ml) IMDM supplemented with 10% (v/v) heat inactivated FCS, penicillin (100 U/ml), streptomycin (100 μg/ml) and IL-2 (20 U/ml; Chiron Benelux). PHA-stimulated PBMC were inoculated with 80 TCID50 virus per 1×10^6^ cells and cultured in IMDM supplemented with 10% (v/v) heat inactivated FCS, penicillin (100 U/ml), streptomycin (100 μg/ml) and IL-2 (20 U/ml). On day 5 fresh PHA-stimulated PBMC were added to the cultures. p24 antigen production in the culture supernatant were determined at day 0, 4, 6 and 8 days after inoculation using an in-house p24 ELISA[3].

**Immunological Analysis**

*Immune phenotyping*

PBMC were used for immune phenotyping. T cell activation was defined as the proportion of cells positive for CD38 and HLA-DR; T cell exhaustion was defined as the proportion of PD1 positive cells; Terminally differentiated T cells were defined as proportion of CD57 positive cells or CD27 and CD28 double negative cells. The following antibodies were used for staining: monoclonal antibody detecting PD-1 (PE) from eBioscience; HLA-DR (FITC), CD38 (PE), CD27 (APCeFluor 780), CD28 (PerCP Cy5.5), CD4+ (PE-Cy7), CD57 (APC), and CD8+ (Pacific BlueCD3 (V500) from BD Biosciences (San Jose, USA). Fluorescence was measured on the FACS Canto II (BD Biosciences). The fractions of cells expressing a marker alone or in combination or the mean fluorescence intensity were determined using FlowJo 7.6 (TreeStar, Ashland, Oregon).

*Intracellular Cytokine Staining*

HIV-specific T cell responses to overlapping peptide pools were assessed in cryopreserved PBMC samples. After thawing, cells were left to rest overnight at 37°C and 5% CO2 in a humidified atmosphere. Subsequently, cells were stimulated with an HIV consensus B Gag peptide pool (2µg/ml final concentration, NIH AIDS Reagent Program) or Staphylococcal enterotoxin B (SEB) in the presence of anti-CD107a-FITC (eBiosciences), brefeldin A and GolgiStop (BD Biosciences), and anti-CD28 and anti-CD29 as costimulation for 6 hours. Cells were washed and stained with anti-CD4+-PECy5.5 (Life Technologies, Waltham, Massachusetts) and anti-CD3-V500 and anti-CD8+-PB (BD Biosciences). Intracellular staining was performed after permeabilization using BD Cytofix/Cytoperm kit, using anti-interleukin 2 (IL-2)–PE, anti-tumor necrosis factor α (TNF-α)–AF700, and anti-macrophage inflammatory protein 1β (MIP-1β)–PECy7 (BD Biosciences), as well as anti-interferon γ (IFN-γ)–APC-AF750 (Life Technologies). Fluorescence was measured on the BD LSR II/Fortessa Flow cytometer (BD Biosciences). The proportion of cytokine-expressing and CD107α positive cells was determined using FlowJo V10 (FlowJo).

*Proliferation Assay*

Proliferation of CD4+ and CD8+ T cells upon antigen stimulation was assessed through the use of the CellTrace™ Violet Cell Proliferation kit (ThermoFisher). Cells were stained with CellTrace Violet according to manufacturer’s protocol (0,5 uM final concentration) and flowcytometry analysis was used to determine that all the cells were labeled with Cell Trace Violet. Subsequently, the cells were stimulated with an HIV consensus B Gag, Env, Pol and Nef peptide pool (2ug/ml final concentration, NIH AIDS Reagent Program). An unstimulated control and positive controls using a peptide pool of CMVpp65 (2ug/ml final concentration, NIH AIDS Reagent Program) or α-CD3 in combination with α-CD28 were included. After 7 days cells were stained with FITC CD3, PerCP-Cy5.5 CD4+ (BD bioscience) and APC CD8+ (BioLegend) for 30 minutes at 4°C. After fixation of the cells with CellFIX (BD) samples were analyzed on the BD FACSCanto™ II to assess the proliferation of CD4+- and CD8+ T-cells under the different conditions. The proportion of proliferating cells and the precursor frequency of HIV-specific T cells was determined using FlowJo V10 (FlowJo).

**HIV specific antibody analysis**

*Luminex assay*

For measuring HIV specific serum IgG, we used stabilized, native-like trimers (SOSIP) of three well-characterized envelope glycoproteins: BG505 SOSIP (clade A) [7], ConM SOSIP (consensus group M) [8] and AMC011 SOSIP (clade B) [9]. To further specify the predominant epitopes targeted, Clade B HIV Env (JRCSF): a gp41 monomer [10], a gp120 monomer consisting of amino acids 31 – 511 (HXB2 numbering) of JRCSF Env with a L111A mutation, and a gp70 scaffold described by Kayman et al. [11] presenting amino acids 291 – 336 in (HXB2 numbering) of JRCSF Env (V3 region) were used. The tetanus toxoid protein (Creative Biolabs) was used as positive control. Serum (1:1000) IgG binding to these antigens was tested in a custom Luminex assay [12] using goat-anti-human IgG-PE (Southern Biotech) as a secondary antibody and the MAGPIX instrument (Luminex). The resulting MFI values are the median of approximately 50 beads per well and were corrected by subtraction of background MFI values (buffer and beads-only wells). A titration of Polyclonal Anti-Human Immunodeficiency Virus Immune Globulin (HIVIG; NIH HIV Reagent Program, Division of AIDS, NIAID, NIH) was used as a positive control, while a pool of HIV negative sera was used as a negative control. IgG binding to the negative control was used as cut-off.

*Neutralization antibodies*

Neutralization assays were performed as described previously [7]. In short, serum was 3-fold serial diluted with a starting dilution of 1:20. Pseudoviruses of the Global Panel of HIV Env Reference Clones [13] were produced in 293T cells and added to the diluted serum for 1 hour at RT. After incubation the mixture was added to pre-seeded TZM-bl reporter cells [14] and incubated for 3 days at 37°C. ID_50_ values were determined as the serum dilution at which infectivity was inhibited by 50%.

**Acknowledgments**

PBMC from participants of The Amsterdam Cohort Studies (ACS) on HIV infection and AIDS were used as controls (chronic HIV infection). ACS is a collaboration between the Public Health Service Amsterdam, the Amsterdam UMC of the University of Amsterdam, Medical Center Jan van Goyen and the HIV Focus Center of the DC-Clinics, are part of the Netherlands HIV Monitoring Foundation and financially supported by the Center for Infectious Disease Control of the Netherlands National Institute for Public Health and the Environment. The following reagents were obtained through the NIH HIV Reagent Program, Division of AIDS, NIAID, NIH: Panel of Global Human Immunodeficiency Virus 1 Env Clones, ARP-12670, from dr. David Montefiori, as well as de HIV subtype B Gag peptide pools, ARP-12425, Nef peptide pool, ARP-12794, Env peptide pool, ARP-6451, Pol peptide pool, ARP-6208, and Human Cytomegalovirus Virus (HCMV) pp65 protein, ARP-11549.

**References**

1. Bosman KJ, Wensing AM, Pijning AE, van Snippenberg WJ, van Ham PM, de Jong DM, et al. **Development of sensitive ddPCR assays to reliably quantify the proviral DNA reservoir in all common circulating HIV subtypes and recombinant forms**. *J Int AIDS Soc* 2018; 21(9):e25185.

2. Hall TA. **BioEdit: a user-friendly biological sequence alignment editor and analysis program for Windows 95/98/NT**. In: *Nucleic acids symposium series*: [London]: Information Retrieval Ltd., c1979-c2000.; 1999. pp. 95-98.

3. van 't Wout AB, Schuitemaker H, Kootstra NA. **Isolation and propagation of HIV-1 on peripheral blood mononuclear cells**. *Nature Protocols* 2008; 3(3):363-370.

4. Lengauer T, Sander O, Sierra S, Thielen A, Kaiser R. **Bioinformatics prediction of HIV coreceptor usage**. *Nat Biotechnol* 2007; 25(12):1407-1410.

5. Björndal A, Deng H, Jansson M, Fiore JR, Colognesi C, Karlsson A, et al. **Coreceptor usage of primary human immunodeficiency virus type 1 isolates varies according to biological phenotype**. *J Virol* 1997; 71(10):7478-7487.

6. Kootstra NA, Munk C, Tonnu N, Landau NR, Verma IM. **Abrogation of postentry restriction of HIV-1-based lentiviral vector transduction in simian cells**. *Proc Natl Acad Sci U S A* 2003; 100(3):1298-1303.

7. Sanders RW, Derking R, Cupo A, Julien J-P, Yasmeen A, de Val N, et al. **A Next-Generation Cleaved, Soluble HIV-1 Env Trimer, BG505 SOSIP.664 gp140, Expresses Multiple Epitopes for Broadly Neutralizing but Not Non-Neutralizing Antibodies**. *PLOS Pathogens* 2013; 9(9):e1003618.

8. Sliepen K, Han BW, Bontjer I, Mooij P, Garces F, Behrens A-J, et al. **Structure and immunogenicity of a stabilized HIV-1 envelope trimer based on a group-M consensus sequence**. *Nature Communications* 2019; 10(1):2355.

9. van Gils MJ, van den Kerkhof TLGM, Ozorowski G, Cottrell CA, Sok D, Pauthner M, et al. **An HIV-1 antibody from an elite neutralizer implicates the fusion peptide as a site of vulnerability**. *Nature Microbiology* 2016; 2(2):16199.

10. Schriek AI, van Haaren MM, Poniman M, Dekkers G, Bentlage AEH, Grobben M, et al. **Anti-HIV-1 Nanobody-IgG1 Constructs With Improved Neutralization Potency and the Ability to Mediate Fc Effector Functions**. *Front Immunol* 2022; 13:893648.

11. Kayman SC, Wu Z, Revesz K, Chen H, Kopelman R, Pinter A. **Presentation of native epitopes in the V1/V2 and V3 regions of human immunodeficiency virus type 1 gp120 by fusion glycoproteins containing isolated gp120 domains**. *Journal of Virology* 1994; 68(1):400-410.

12. Grobben M, van der Straten K, Brouwer PJM, Brinkkemper M, Maisonnasse P, Dereuddre-Bosquet N, et al. **Cross-reactive antibodies after SARS-CoV-2 infection and vaccination**. *eLife* 2021; 10:e70330.

13. deCamp A, Hraber P, Bailer RT, Seaman MS, Ochsenbauer C, Kappes J, et al. **Global Panel of HIV-1 Env Reference Strains for Standardized Assessments of Vaccine-Elicited Neutralizing Antibodies**. *Journal of Virology* 2014; 88(5):2489-2507.

14. Sarzotti-Kelsoe M, Bailer RT, Turk E, Lin CL, Bilska M, Greene KM, et al. **Optimization and validation of the TZM-bl assay for standardized assessments of neutralizing antibodies against HIV-1**. *J Immunol Methods* 2014; 409:131-146.
