# Supplementary material for: Virological and immunological correlates of HIV posttreatment control after temporal antiretroviral therapy during acute HIV infection
Source: AIDS. 2023 Sep 11;37(15):2297–304. doi: 10.1097/QAD.0000000000003722 (PMC10653294; doi:10.1097/QAD.0000000000003722)
Supplement: Supplemental Digital Content [file aids-37-2297-s003.docx]

**Supplementary Table 1.**

**Characteristics of participants from ACS with untreated chronic HIV infection**

|  | **Immune phenotyping** | **T cell functionality** |
| --- | --- | --- |
|  | number or median (IQR) | number or median (IQR) |
| n | 12 | 15 |
| male | 12 | 15 |
| HIV positive at entry | 7^$^ | 15 |
| Month after entry or seroconversion^#^ | 40,6 (33,8-68,6) | 40,6 (29,4-50,1) |
| CD4+ T cell count (x10^9^/L) ^#^ | 0,47 (0,29-0,51) | 0,43 (0,36-0,46) |
| Viral load (LOG copies/ml) ^#^ | 4,3 (4,0-4,7) | 4,1 (3,5-4,7) |

^$^ 5 participants seroconverted for HIV during follow up

^#^ time point of analysis

**Supplementary Table 2. Global Panel of Viruses**

| Virus | Clade | Tier | IC50 |
| --- | --- | --- | --- |
| 398F1 | A | 1 | <20 |
| X2278 | B | 2 | <20 |
| CNE8 | CRF01 | 2 | <20 |
| TRO.11 | B | 2 | <20 |
| Ce1176_A3 | C | 2 | <20 |
| 25710-2.43 | C | 2 | <20 |
| Ce703010217_B6 | C | 2 | <20 |
| X1632-S2-B10 | G | 2 | <20 |
| BJOX002000.03.2 | CRF07 | 2 | <20 |
| 246-F3_C10_2 | AC | 2 | <20 |
| CH119.10 | CRF07 | 2 | <20 |
